# Isolated diffuse choroidal hemangioma without systemic symptoms: a case report

**DOI:** 10.1186/s12886-023-03057-2

**Published:** 2023-07-06

**Authors:** Xiaohua Zhang, Yongping Hu, Di Li, Xiaoxiao Qian, Yongning Xu, Man Guo, Qihang Li

**Affiliations:** Department of Ophthalmology, Affiliated Hangzhou First People’s Hospital, Zhejiang University School of Medicine, Hangzhou, China

**Keywords:** Case report, Sturge-Weber syndrome, Choroidal haemangioma, Abnormal conjunctival vessels, Abnormal episcleral vessels

## Abstract

**Background:**

Sturge-Weber syndrome is a nonhereditary congenital neurocutaneous syndrome characterized by a distinctive facial capillary malformation,neurological abnormalities, and ocular abnormalities such as glaucoma and choroidal hemangioma.It can be divided into different subtypes according to different clinical manifestations. It is rare for a patient to present with isolated difuse choroidal hemangioma and ipsilateral abnormal conjunctival and episcleral vessels without other systemic symptoms.

**Case presentation:**

We report a 30-year-old man with isolated diffuse choroidal hemangioma in his right eye without systemic symptoms, such as vascular malformations in the skin or leptomeningeal angiomatosis. The only additional ophthalmic finding was ipsilateral abnormal conjunctival and episcleral vessels without glaucoma. However, there was no evidence of leptomeningeal angiomatosis or port-wine stain on the right side of the face, or glaucoma, which are common clinical manifestations of the Sturge-Weber syndrome (SWS).The absence of these characteristic symptoms did not preclude the diagnosis, and the patient could be diagnosed with a particular subtype of SWS.

**Conclusions:**

This is a rare case of documented isolated difuse choroidal hemangioma with ipsilateral abnormal conjunctival and episcleral vessels without glaucoma which we think it is belonging to a particular subtype of SWS.In addition to the traditional clinical manifestations, more and more atypical clinical manifestations are also accompanied by SWS, which requires our clinicians to continuously discover and report, so as to help more clinicians understand this disease.

## Background

Sturge-Weber syndrome (SWS), also called encephalo-trigeminal angiomatosis, is a rare, sporadic phacomatosis with no gender predilection, nor heritability. Its clinical presentations mainly include leptomeningeal angiomatosis, classic ipsilateral facial nevus flammeus, and ocular abnormalities, which can involve eyelid, bulbar conjunctiva, sclera, iris, anterior chamber, choroid, and retina [1–4]. The eyes are involved in more than 50% of SWS patients, and the main manifestations are glaucoma and diffuse choroidal hemangioma (DCH) [5]. Choroidal hemangioma can present as either circumscribed or diffuse. The DCH is typically associated with SWS, while circumscribed choroidal hemangioma (CCH) may be observed in SWS patients [6]. DCH is a benign vascular tumor that its typical lesions often involve more than half of the fundus with ill-defined borders. The lesion may be visually symptomatic at any age for changes in subfoveal choroidal thickness that may cause retinal defocus, refractive error, and hyperopic amblyopia. Later stages may include foveal distortion, photoreceptor damage or secondary exudative retinal detachment or glaucoma.

The embryologic basis of SWS has been reported to be related to an impaired development of the cell precursors in the neural crest during the first embryological trimester, leading to the characteristic malformations observed in the central nervous system, skin, and eyes. During early stage 2 to 3 of vascular development in the first trimester, the primitive vascular system divides into the external portion that feeds and drains the facial skin and scalp, a middle portion investing the meninges, and the deep portion that feeds and drains the brain [7–9].

A variety of chromosomal abnormalities has been reported inassociation with Sturge-Weber syndrome 16,17; however, somatic mutation has most recently been cited as the probable cause of Sturge-Weber syndrome, given the sporadic occurrence of localized, asymmetric abnormality of blood vessel formation. The timing and location of the somatic mutation is important in presentation [10–12].

Roach et al. classified SWS into three types: Type I (the most common) that includes leptomeningeal and facial angioma with or without glaucoma; type 2 presents with facial angioma as the most prominent manifestation with or without glaucoma, without brain involvement; type 3, the rarest type, that is accompanied by leptomeningeal angioma [13]. SWS is a systemic disease that is mainly diagnosed via ancillary tests with multimodal imaging. The present study aimed to report a case with an isolated DCH without systemic symptoms, and the only important ophthalmic finding was ipsilateral abnormal conjunctival and episcleral vessels, which did not meet the Roach et al.’s classification. However, based on embryological data, DCH with conjunctival and episcleral abnormalities could also be diagnosed as SWS.

## Case presentation

**Fig. 1:**
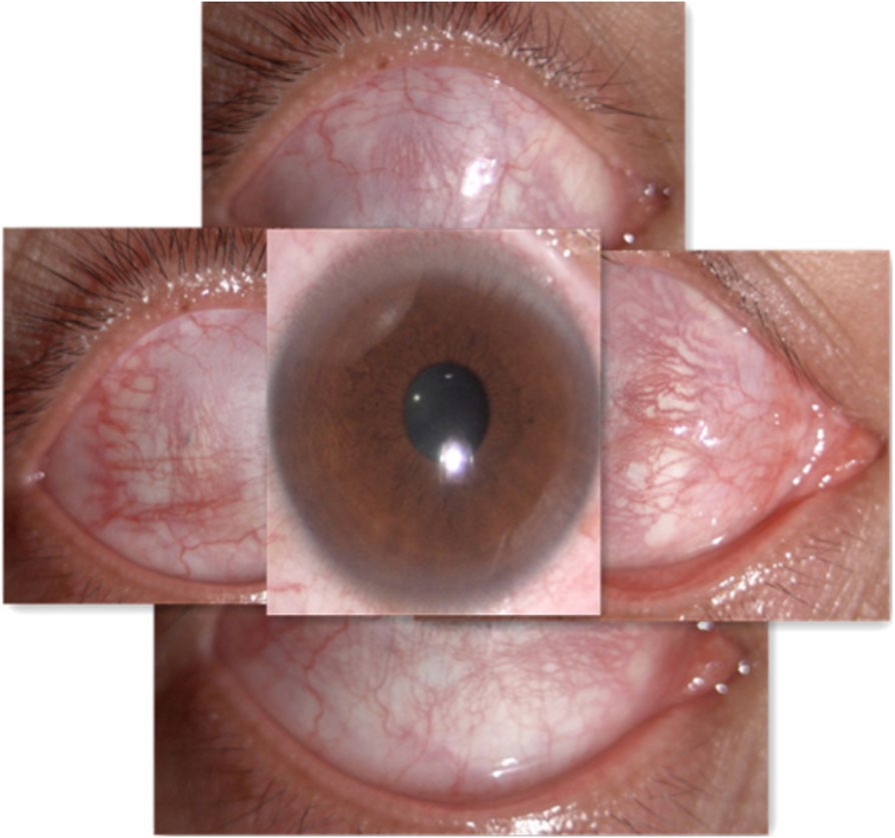
Slit lamp images of the anterior segment in the RE. Slit lamp examination showed vascular in the conjunctiva or sclera was brushlike dilation, proliferation, and some were hemangiomatous

**Fig. 2:**
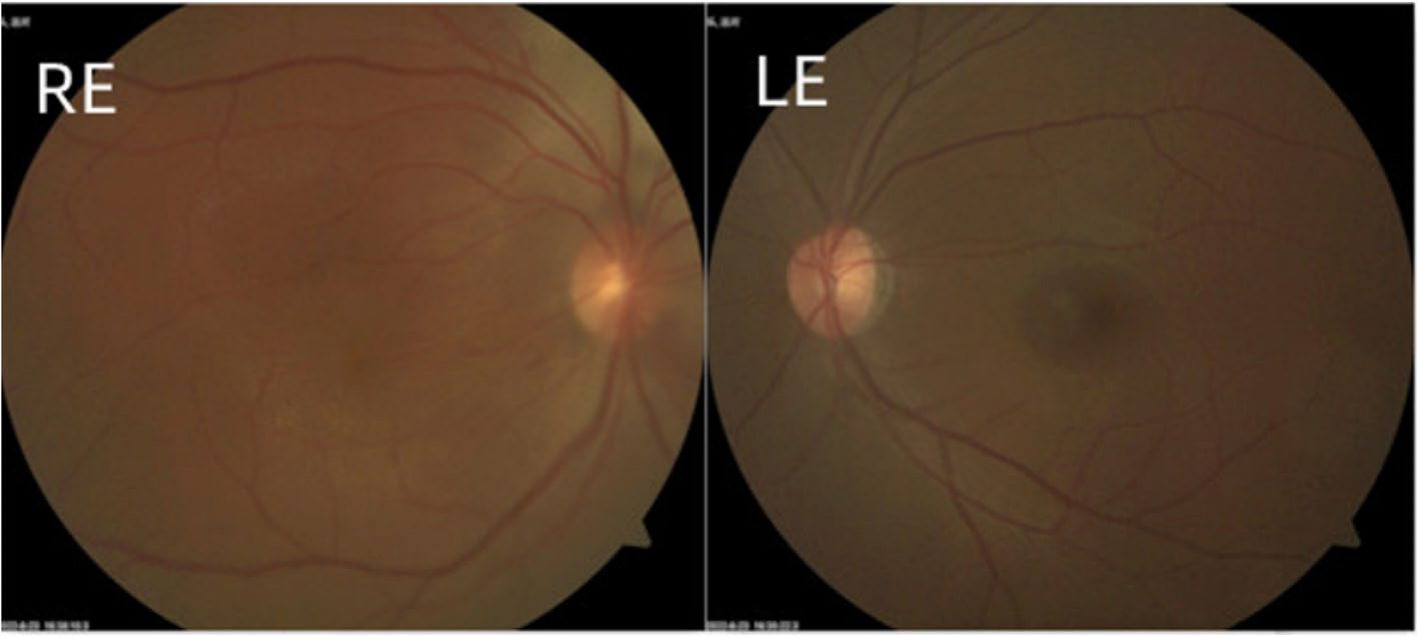
Fundus color photographs of the RE and LE. The image in the right fundu is darker and shows diffuse deep red colour with respect to the fellow eye where there is scattered with yellow-white lesions on fundus posterior pole, involving the macular area

**Fig. 3:**
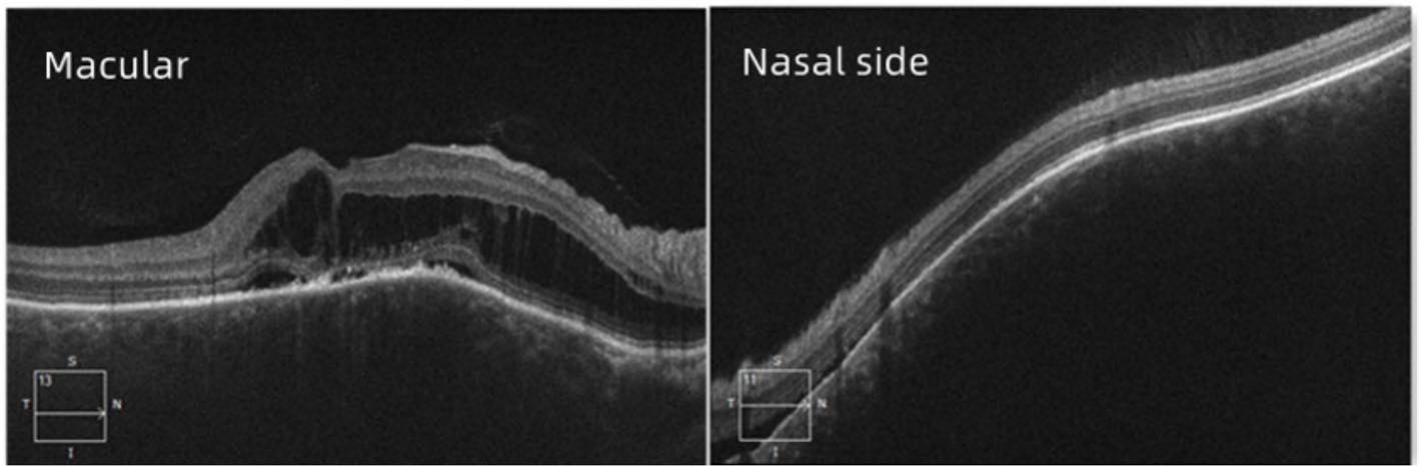
Enhanced depth imaging (EDI) spectral domain optical coherence tomography (SD-OCT) images of the RE.SD-OCT shows RE diffuse choroidal thickening, and diffuse elevation of the retinal pigment epithelial layer with a dome-shaped, especially in the nasal side. There is cystic edema in the retinal neuroepithelium layer, subretinal liquid cavities in the fovea and pigment epithelium hyperplasia is also observed

**Fig. 4:**
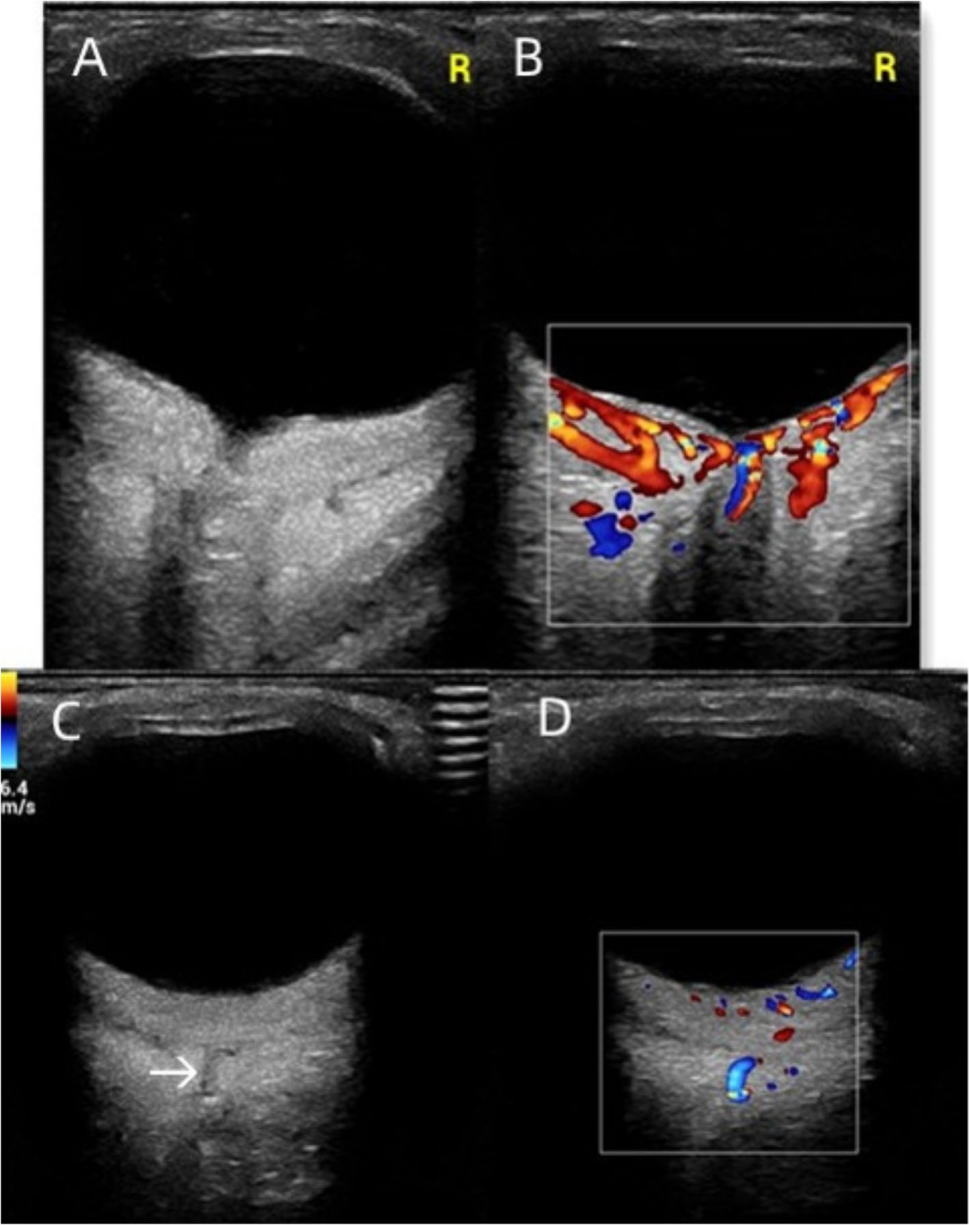
Ultrasound scans of the RE. **A** Ultrasonography revealed a diffusely thickened choroid with increased internal re-flectivity. **B** Color Doppler ultrasonography showed enhanced choroid blood flow signal. **C** Ultrasound examination revealed a tubular area of low echo in the orbit, which was the dilated ophthalmic vein (arrows). **D** Color Doppler showed blood flow signals in the tubular area

**Fig. 5:**
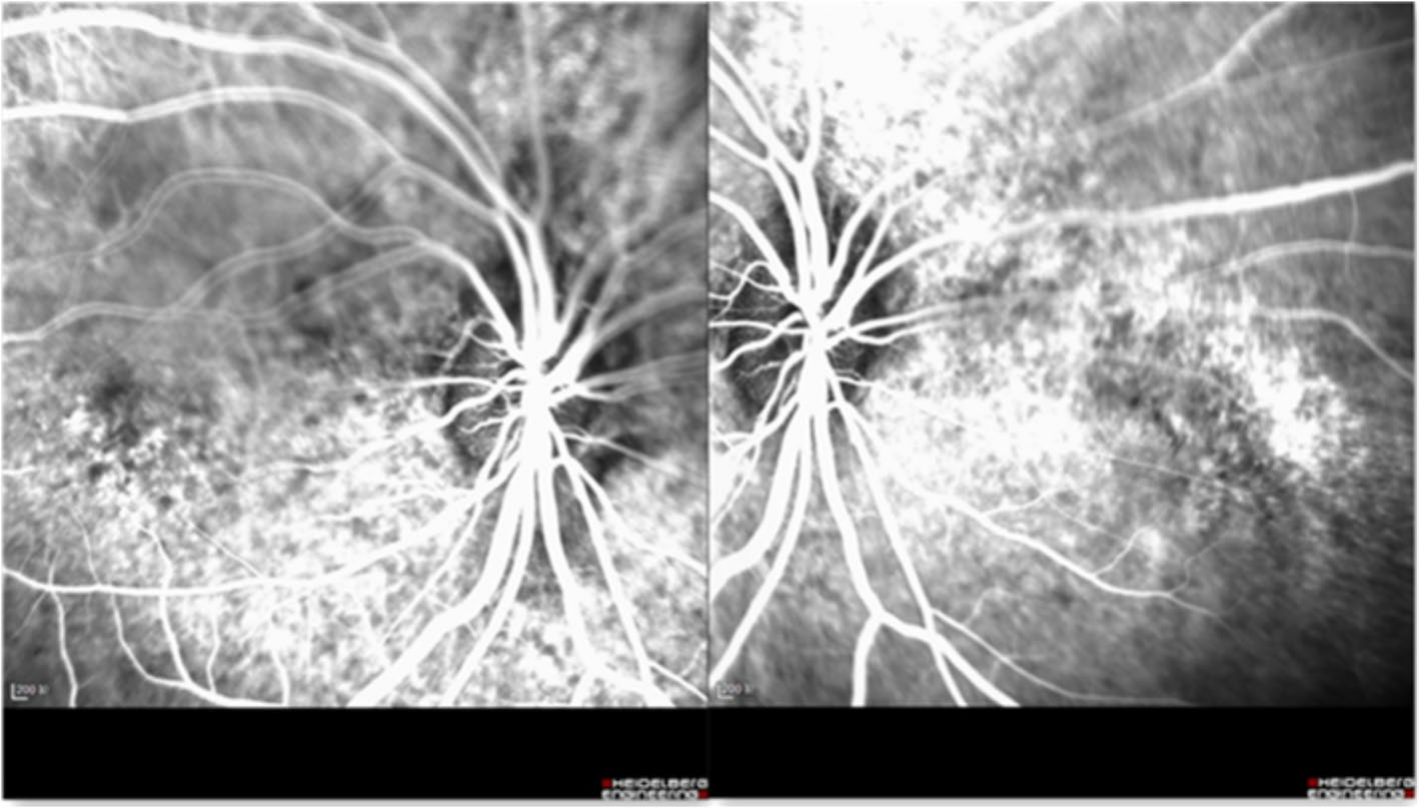
ICGA photography of RE. ICGA revealed the posterior pole of choroid was filled with a network of small-caliber vessels

**Fig. 6:**
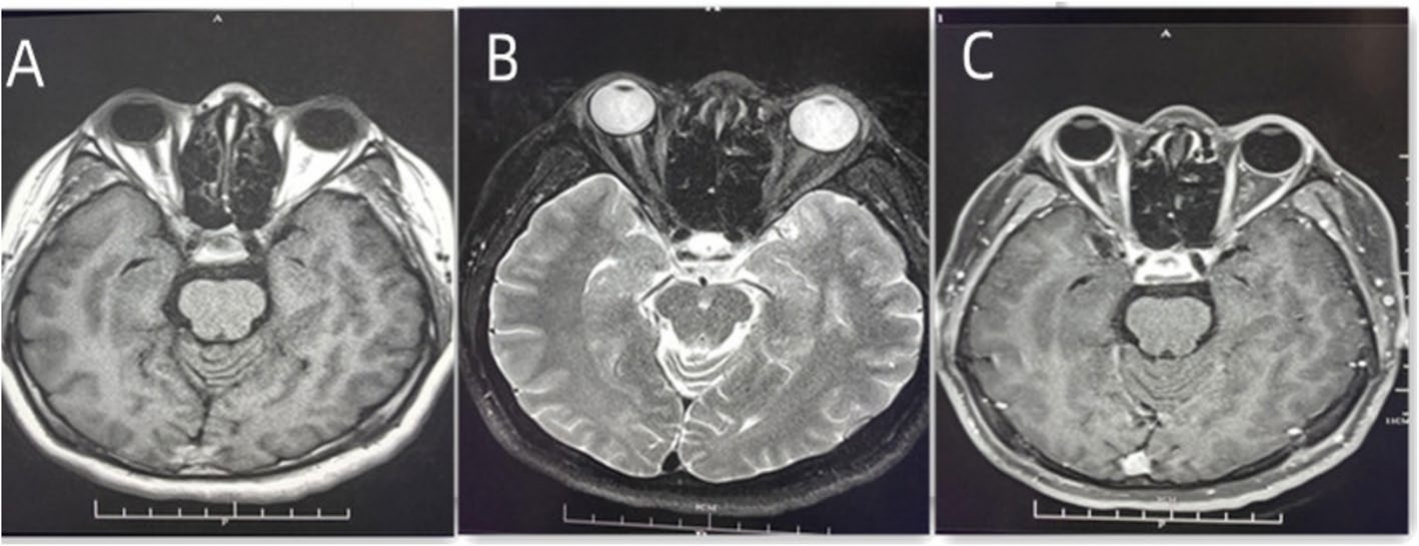
Orbital MRI scan images. **A** Orbital MRI scan suggested the anterior edge of the posterior wall of the RE was thickened, with slightly higher signal on T1WI. **B **Orbital MRI scan suggested the anterior edge of the posterior wall of the RE was slightly lower signal on T2W1. **C** Enhanced scan showed obvious uniform enhancement compared with the LE

A 30-year-old man was admitted to our ophthalmology department due to red eye and blurred vision in the right eye (RE) for more than 1 year. No nevus flammeus was observed in the eyelid or skin, and he also denied undergoing laser surgery. Magnetic resonance imaging (MRI) finding did not confirm leptomeningeal hemangioma. Intraocular pressure was 16.3 and 16.8 mmHg in the RE and the left eye (LE), respectively. Best corrected visual acuity (BCVA) was 0.2 and 1.0 in the RE and LE, respectively. Slit lamp examination showed diffused pinkish discoloration related to the increased conjunctival and episcleral vascularization. Vascular in the conjunctiva or sclera showed brush-like dilation, proliferation, and occasionally was hemangiomatous (Fig. 1). The iris was brown, with no abnormal neovascularization or hyperpigmentation. Gonioscopy revealed that the angle was open in both eyes. The pupil showed a brilliant red reflex in the RE in contrast to the normal reflex in the opposite pupil. The presence of a diffuse red-orange color appearance (tomato catsup) of the fundus with respect to the LE was detected by ophthalmoscopy. There were scattered, yellow-white lesions on posterior pole of the fundus, involving the macular area (Fig. 2). Enhanced depth imaging (EDI) using spectral-domain optical coherence tomography (SD-OCT) of the RE showed diffuse choroidal thickening, and diffuse elevation of the retinal pigment epithelium (RPE) with a dome-shaped, especially around the optic disc. In some areas, the choroid-scleral junction was out of the range of the instrument and could not be measured due to the technical limitation of the instrument. There was cystic edema in the neuroepithelial layer of retina, and subretinal liquid cavities in the fovea and RPE hyperplasia were also observed (Fig. 3). Color Doppler ultrasound displayed placoid choroidal thickening with increased internal reflectivity, from the posterior pole to the peripheral choroid, especially around the optic disc. In the color mode, it can be observed that the choroid blood supply is very rich, presenting a high-speed and low resistance spectrum. At the same time, the superior ophthalmic vein is dilated (Fig. 4). The patient underwent indocyanine green angiography (ICGA) on the same day. ICGA revealed rapid filling of the diffuse choroidal hemangioma’s vascular network in the early stages. The vessels were fine, lacy and showed a web configuration, and were diffusely distributed over the posterior pole (Fig. 5). Orbital MRI revealed that the anterior edge of the posterior wall of the RE was thickened, with a slightly higher signal-intensity on T1-weighted image (T1WI) and a slightly lower signal-intensity on T2WI. Enhanced scan showed obvious uniform enhancement compared with the LE (Fig. 6). Contrast-enhanced scan showed an obviously uniform enhancement in the RE compared with the LE. Contrast-enhanced cranial MRI illustrated no obvious abnormality. The final diagnosis was DCH in the RE, conjunctival and scleral hemangioma, which would be assumed as a variant of SWS.

## Discussion and conclusions

According to the histopathological observations, choroidal hemangiomas are classified according to the type of vessels within the tumor as cavernous, capillary, or mixed [14]. Several studies showed that choroidal hemangiomas are non-proliferative tumors as the endothelial cells lining their vascular channels do not proliferate, however, these tumors progressively enlarge secondary to venous congestion within the mass rather than cell proliferation [14, 15]. Bichsel et al. found that the abnormal choroidal vessels in SWS are akin to a capillary malformation of the choroidal blood vessels, because they share the same R183Q causative somatic mutation in *GNAQ* gene [16, 17]. A number of researchers demonstrated that the term “choroidal hemangioma” is not appropriate and should be substituted by “choroidal capillary malformation” [18]. According to embryology, the V1 branch of the trigeminal nerve and the capillary plexuses are derived from neural crest cells. Vascular endothelial growth factor (VEGF) is largely produced to attract vascular plexuses when the ophthalmic branch of the trigeminal nerve starts to develop from neural crest cells. This process continues until they reach their final location. The vessel-trigeminal-ectoderm complex finally develops into the skin of the upper face, upper eyelid, and conjunctival vessels, which are ultimately innervated by the trigeminal nerve (V1). Some vessels coalesce in the inner part of the optic placode and become choriocapillaris [19–21]. In recent years, it has been repeatedly reported that local abnormal vascularization in embryos is caused by somatic mutation in early embryonic development, and it has been confirmed that somatic mutation of *GNAQ* gene, which leads to microvascular malformation, is the same pathogenic gene related to SWS [22]. Jasmine et al. found that solitary choroidal hemangioma (SCH) and DCH both exhibit mutations in *GNAQ* gene, while at different codons, including DCH at R183Q, which is consistent with SWS, and SCH at Q209 [16]. The clinical manifestations vary depending on where and when the mutations occur. In the embryo, the larger the affected vascular sheet, the greater the extent of the affected area. If the somatic mutations occur early, abnormal vascular sheet originates before the primary meningeal layer migrates, all the neural, ocular, and cutaneous will be involved. In contrast, if the abnormal vascular sheet forms more anteriorly, then the affected area will be smaller. Hansell Soto et al. demonstrated that if the abnormal vascular sheet originates after the migration of primary meningeal layer, SWS type II may be manifested [23]. Based on the upper eyelid skin and conjunctival vessels arising from a common vascular sheet, vascular angiomatosis of upper eyelid skin and conjunctiva would phenotypically be equivalent and would be present or absent, independent of each other. SWS type II can be subdivided into type IIa if the skin is involved or type IIb if only conjunctival telangiectasia is present [23]. Thus, in the present study, SWS type II, a phenotypic variant of SWS, could be considered. Plateroti et al. reported a rare case of SWS with bilateral nevus flammeus and ocular melanocytosis of the right iris, which accompanied with the presence of ipsilateral iris mammillations, glaucoma, and DCHS [24]. The appearance of ipsilateral iris pigment abnormality in this SWS patient is incidental, or is it a special and rare SWS-related manifestation? Fischbein et al. reported a case who received gadolinium, and there was no evidence of leptomeningeal enhancement [25]. Amirikia et al. reported bilateral choroidal hemangiomas associated with unilateral facial nevus flammeus in a SWS patient [26]. Scott et al. reported the first case of SWS with DCH in one eye and CCH in his fellow eye [6]. After a comprehensive literature review, it was revealed that there are other clinical symptoms associated with SWS patients. As more cases are identified and underlying mechanisms are figured out, the diagnostic criteria for SWS will be accordingly updated and generalized.

According to the consensus guidelines published in 2021 regarding management of SWS, every patient diagnosed with SWS or with a high-risk facial port-wine birthmark (PWB),or ocular vascular anomalies involving the eyelids should be referred to a pediatric ophthalmologist for a baseline eye evaluation and have periodic follow-up, because of the risk of preventable visual loss. While glaucoma is a common ocular complication of SWS. The treatment of glaucoma in patients differs based on the age and clinical presentation of the patient. High pressure or wide intraocular pressure fluctuations, result in an irreversible optic neuropathy and subsequent progressive visual impairment. Thus, early detection and treatment is vital [27]. The glaucoma can present in infancy (0–3 years) and others that present later in life. In young children, the disease is almost always treated surgically, followed by medications and/or laser. The glaucoma presenting in older individuals may first be managed with medications, either topical or systemic. Surgery or laser treatment is often necessary if medications are not sufficient to control the disease [28, 29].

The treatment of DCH aims to induce involution of the hemangioma, reduce tumor size, induce vessel atrophy, decrease the volume of subretinal and intraretinal fluid, and minimize the serous detachment of neurosensory retina. DCH may be asymptomatic, or cause visual loss secondary to refractive error. Such patients can be followed up, and hyperopic refractive errors can be treated with refraction, corrective lenses, and amblyopia therapy. Once complications (e.g., serous retinal detachment and subretinal hemorrhage) appear, which can lead to grave loss of vision, treatments should be urgently initiated. In the current study, the patient presented with a significant vision loss. SD-OCT revealed macular cystoid edema and subretinal fluid. RPE hyperplasia was also observed. Therefore, this patient had indications for treatment. Radiotherapy, photodynamic therapy, and transpupillary thermotherapy are currently the preferred therapeutic methods for such patients. In addition to RPE hyperplasia, a previous study demonstrated that the outer retinal layers were thinner in SWS patients with choroidal hemangioma [30]. Because the choroid is fundamental for the outer five layers of the retina. Indeed, choroidal thickness changes have been implicated in retinal layer alterations, especially of the outer retinal layers. From this case, we also learned that the tomato catsup appearance requires a true color fundus camera, otherwise it is easy to miss important information.

We have described a rare case of isolated difuse choroidal hemangioma with ipsilateral abnormal conjunctival and episcleral vessels without glaucoma which we think it is belonging to a particular subtype of SWS. Diagnosis and evaluation of SWS can be facilitated with multimodal imaging techniques. In addition to the traditional clinical manifestations, more and more atypical clinical manifestations are also accompanied by SWS, which requires our clinicians to continuously discover and report, so as to help more clinicians understand this disease.

## Data Availability

All data generated or analysed during this study are included in this published article.

## Abbreviations

SWS: Sturge-Weber syndrome
DCH: Diffuse choroidal hemangioma
CCH: Circumscribed choroidal hemangioma
RE: Right eye
MRI: Magnetic resonance imaging
LE: Left eye
BCVA: Best corrected visual acuity
EDI: Enhanced depth imaging
SD-OCT: Spectral-domain optical coherence tomography
RPE: Retinal pigment epithelium
ICGA: Indocyanine green angiography
T1WI: T1-weighted image
VEGF: Vascular endothelial growth factor
SCH: Solitary choroidal hemangioma
